# MerTK mediates dexamethasone-induced suppression of antiviral CD8^+^ T cell immunity and exacerbates chronic LCMV infection

**DOI:** 10.3389/fcimb.2026.1961552

**Published:** 2026-10-21

**Authors:** Tom Adomati, Thamer A. Hamdan, Barbara Seliger, Vikas Duhan, Jacqueline E. Akite, Herbert Ariaka, Hilal Bhat, Murtaza Ali, Lamin B. Cham

**Affiliations:** 1 Laboratory of Experimental Medicine and Paediatrics, Faculty of Medicine and Health Sciences, University of Antwerp, Antwerp, Belgium; 2 Department of Gastroenterology and Hepatology, University Hospital Antwerp, Edegem, Belgium; 3 Institute of Pathology, Section Immunopathology, Martin Luther University Halle-Wittenberg, Halle (Saale), Germany; 4 Institute of Translational Immunology, Faculty of Health Sciences, Brandenburg Medical School, Brandenburg an der Havel, Germany; 5 Frazer Institute, The University of Queensland, Woolloongabba, QLD, Australia; 6 African Centre for Systematic Reviews and Knowledge Translation, Makerere University, Kampala, Uganda; 7 Department of Cardiac Surgery, Uganda Heart Institute, Kampala, Uganda; 8 The National Center for Radiation Research in Oncology, Dresden, Germany; 9 Medizinische Klinik und Poliklinik Zentrum für Innere Medizin, Hamburg, Germany; 10 Department of Microbiology, Immunology and Infectious Diseases, College of Medicine and Health Sciences, Arabian Gulf University, Manama, Bahrain

**Keywords:** CD8^+^ T cell-suppression, dexamethasone, glucocorticoids, IL-10, lymphocytic choriomeningitis virus (LCMV), MertkMerTK, PD-1, pleiotropism

## Abstract

**Introduction:**

Glucocorticoids (GCs) such as dexamethasoneare widely used to treat allergies, autoimmune disorders, and chronic inflammatory conditions. However, their impact on antiviral immunity is complex and often contradictory. Clinically, dexamethasone can be beneficial in severe viral infections by suppressing excessive inflammation and limiting tissue injury. In contrast, during mild, early, or chronic infections, its immunosuppressive activity may delay viral clearance, promote viral replication or reactivation, and ultimately worsen disease outcomes. Reports describing both reduced and enhanced viral replication following GC treatment underscore the pleiotropic nature of GC action, yet the underlying mechanisms remain poorly understood.

**Methods:**

We investigated the effect of dexamethasone during persistent LCMV infection in wild-type (*Mertk^+/+^*) and MerTK-deficient (*Mertk^–/–^*) mice (20-25g body weight). Animals were infected with 2 × 10^4^ PFU of the LCMV Docile strain intravenously and randomly assigned to two intervention arms, receiving either PBS or dexamethasone (2.5mg/kg, dose adjusted according to body weight) intraperitoneally every 48 hours.On day 8 post-infection, blood was collected prior to euthanasia, after which spleen and liver samples were harvested for flow-cytometric analysis. Viral titres were subsequently quantified in the spleen, liver, kidney, lung, and lymph nodes.

**Results:**

In *Mertk^+/+^* mice, dexamethasone induced pronounced loss of CD8^+^ T cells and enhanced MerTK-dependent suppression of virus-specific CD8^+^ T cell responses, an effect largely absent in *Mertk^–/–^* animals. Functionally, dexamethasone substantially increases production of the anti-inflammatory cytokine IL-10 by macrophages and dendritic cells and elevates expression of the inhibitory checkpoint molecule PD-1 on LCMV-specific CD8^+^ T cells in *Mertk^+/+^* mice. Moreover, expression of KLRG1, CD44, and CD127 on CD8^+^ T cells were substantially reduced, indicating impaired effector differentiation, whereas CD62L^+^ CD8^+^ T cells were upregulated, suggesting retention in a naïve or central-memory–like state. Together, these changes promoted functionally impaired CD8^+^ T cell phenotype, weakened LCMV-specific immunity, and facilitated viral dissemination across multiple organs examined. Genetic ablation of MerTK mitigated these immunosuppressive effects, resulting in significantly improved viral control.

**Discussion:**

These findings demonstrate that the immunosuppressive effects of dexamethasone are critically dependent on the MerTK-mediated immunoregulatory axis. By identifying MerTK as a central node through which GCs reinforce inhibitory immune circuits, our study highlights MerTK as a potential target for future therapeutic intervention at the frontier of viral infections and inflammatory diseases. However, this therapeutic potential is currently supported solely by genetic loss-of-function evidence, and future studies employing pharmacological MerTK inhibition will be required to validate its efficacy and translational applicability.

## 1 Introduction

Glucocorticoids (GCs) are potent anti-inflammatory and immunosuppressive drugs widely used to treat inflammatory, allergic and autoimmune diseases (Almawi et al., 1996; Ayroldi et al., 2012; Kunicka et al., 1993; Rolfe et al., 1992) as well as to reduce mortality in severe COVID-19 (Knoll et al., 2024). They exert their anti-inflammatory effects by limiting vasodilation and immune-cell recruitment, and by crossing cell membranes to bind cytoplasmic GC receptors, thereby suppressing pro-inflammatory cytokine production through transcriptional downregulation (Patel et al., 2020). However, dexamethasone treatment is a double-edged sword. Although highly effective across a wide range of inflammatory and immune-mediated conditions, its long-term or systemic use is associated with substantial adverse effects including the promotion of osteoblast apoptosis through multiple signalling pathways (Deng et al., 2019; Ianiro et al., 2020; Xing et al., 2019). GC-induced apoptosis remains a biologically important and clinically relevant process (Brewer et al., 2002), selectively depleting CD4^+^ effector T cells while sparing Tregs (Chen et al., 2004). A distinctive feature of murine and human B-1 cells is their constitutive production of the anti-inflammatory cytokine IL-10, which also functions as an autocrine growth factor (Griffin and Rothstein, 2012; Ishida et al., 1992). While dexamethasone depletes B-2 cells, B-1 cells are spared, and these surviving B-1 cells retain and even enhance IL-10 production and secretion (Chen et al., 2014).

From a virological perspective, using corticosteroids to control excessive inflammation during viral infection presents a distinct trade-off: while these drugs can suppress damaging immune responses and improve symptoms in some models, they can simultaneously impair antiviral immunity, potentially allowing the virus to persist or replicate more freely in others. For instance increased viral replication following dexamethasone treatment has been reported in cultured mammalian cells infected with HSV-1 (Du et al., 2012; Hara et al., 2009), MMTV (Indik et al., 2007; Parks et al., 1974), and other retroviruses (Solodushko et al., 2009). In contrast, Lancz et al. observed that dexamethasone treatment reduced the replication of VSV in L929 cells (Lancz et al., 1990). These divergent outcomes highlight the context-dependent nature of GCs and underscore that their precise mechanisms of action remain incompletely understood. Our previous work using a cytopathic VSV model, a virus that induces rapid and robust cell death showed that dexamethasone exacerbates viral burden by suppressing innate antiviral immunity. Specifically, dexa treatment led to elevated IL-10, TGF-β, and SOCS1/3 expression in wild-type (*Mertk^+/+^*) mice and this weakening of antiviral defences worsens infection. These effects were largely abrogated in MerTK-deficient (*Mertk^–/–^*) mice (Adomati et al., 2020), identifying MerTK as a central mediator of GC-induced immunosuppression.

MerTK is a receptor expressed on macrophages and dendritic cells (DCs) that typically binds phosphatidylserine (PS) on apoptotic cells through its ligands GAS6/PROS1 (Mark et al., 1996; Nagata et al., 1996; Ohashi et al., 1995; Stitt et al., 1995) thereby suppressing inflammation and maintaining immune tolerance (Scott et al., 2001; Sen et al., 2007; Zizzo et al., 2012). Beyond clearing dead cells, MerTK signalling diminishes T-cell antigen-scanning sensitivity, enabling mononuclear phagocytes to protect human islets from autoimmune attack, while tumor-associated macrophages exploit this pathway to block tumor-specific T-cell arrest and promote immune evasion. Conversely, MerTK inhibition amplifies T-cell activation and can rapidly precipitate autoimmune pathology, including accelerated type 1 diabetes (Lindsay et al., 2021). In HCC, the MerTK axis also confers resistance to anti-PD-1/PD-L1 therapy by limiting ferroptosis (Wang et al., 2024). In chronic lymphocytic choriomeningitis virus (LCMV) infection, we recently demonstrated that elevated burden of apoptotic cells together with increased MerTK expression correlates with weakened antiviral CD8^+^ T-cell responses and poor viral control. Notably, genetic ablation of MerTK restores robust CD8^+^ T cell immunity, resulting in efficient viral clearance (Adomati et al., 2026). Together, these functions position MerTK as a compelling target for viral and cancer immunotherapy, autoimmune modulation, and tolerance engineering.

In this study, we discovered that dexamethasone promotes MerTK-driven suppression of CD8^+^ T cell immunity, accompanied by substantial CD8^+^ T cell loss, profoundly weakening antiviral responses during LCMV infection, an effect largely eliminated in MerTK-deficient mice. Dexamethasone markedly increases IL-10 production by antigen-presenting cells (APCs) and elevates PD-1 expression on CD8^+^ T cells, promoting dysfunctional effector differentiation. Together, these alterations drive functional decline, diminish antiviral defence, and exacerbate viral replication. In the absence of MerTK, these immunosuppressive effects are largely lost, resulting in markedly improved LCMV clearance across both treatments. Overall, our findings reveal that dexamethasone can intensify chronic infection, with MerTK emerging as a key mediator of GC-potentiated suppression and a promising target for modulating inflammation for therapeutic intervention.

## 2 Materials and methods

### 2.1 Ethics statement

Animal experiments received authorization by the Veterinary Office of North Rhine-Westphalia (Veterinäramt Nordrhein-Westfalen; Düsseldorf-Essen, Germany) under license number 2016.A289.G1569 (State Agency for Nature, Environmental and Consumer Protection of North Rhine-Westphalia (Landesamt für Natur, Umwelt und Verbraucherschutz Nordrhein-Westfalen). Procedures were also conducted in accordance with the German laws for animal protection or according to institutional guidelines at the Ontario Cancer Institute of the University of Toronto Health Network. All animal care and protocols adhered to German animal protection laws (Tierschutzgesetz) and European laws (Directive 2010/63/EU) and Federation of European Laboratory Animal Science Associations (FELASA; http://www.felasa.eu/) guidelines. Animals were euthanized by cervical dislocation in accordance with approved protocols.

### 2.2 Mice

*Mertk^–/–^* mice were maintained by heterozygous breeding on a mixed Sv129 × C57BL/6 (B6) background. *Mertk*^−/−^ mice were backcrossed for at least five generations onto the C57BL/6 background and were directly compared with littermate *Mertk*^+/+^ controls. Both male and female mice aged 7–10 weeks (20–25 g body weight) were included in all experiments.

### 2.3 Virus and focus formation assay

LCMV strains docile were originally obtained from F. Lehmann-Grube (Heinrich Pette Institute, Hamburg, Germany) and propagated on L929 cells (obtained from ATCC, NCTC clone 929). LCMV-docile was donated by Prof. Dr. R. Zinkernagel (University of Zurich, Zurich, Switzerland). Mice were infected intravenously (i.v.) with 2×10^4^ plaque-forming units (PFU) of LCMV-docile. LCMV viral titres were quantified by a focus-forming assay on MC57 fibroblasts, as previously described (Battegay et al., 1991). In brief, organs were homogenized in DMEM containing 2% fetal calf serum (FCS), serial diluted 1:3 over 12 steps in 96-well round-bottom plates, and plaqued on MC57 cells. After incubation for 3 h at 37 °C, an overlay consisting of a 1:1 mixture of methyl cellulose and Iscove’s modified Dulbecco’s medium was added. Plaques were counted 48 h later via LCMV nucleoprotein (NP) staining. For staining, cells were fixed with 4% formaldehyde solution in PBS, permeabilized with 1% Triton-X solution in PBS, blocked with 10% FCS in PBS, and stained with rabbit anti-LCMV NP Ab (made in-house) as primary Antibody. HRP-conjugated anti-rabbit-IgG Ab (from Jackson ImmunoResearch, *RRID:* AB_2339647) was used as a secondary Antibody (diluted 1:400 to the original concentration in distilled water). Plaques were detected by chemiluminescence (0.2 M Na_2_HPO_4_ + 0.1 M citric acid + 30% H_2_O_2_ + o-phenylenediamine dihydrochloride (OPD, Sigma, P8412); all other chemicals were from Sigma-Aldrich, Saint Louis, MO, USA).

### 2.4 Genotyping by PCR

Genomic DNA was isolated from tail biopsies. Tail samples were digested overnight in lysis buffer containing proteinase K, and DNA was extracted using the phenol–chloroform method (Javadi et al., 2014). Genotyping of the murine MerTK locus was performed using the following oligonucleotide primers (biomers.net, Ulm/Donau, Germany): 1) *Mertk^+/+^* primer: 5′-TTC TTG TTC TGG GGT TGA CTC-3′; 2) Common sense primer: 5′-CAT CTG GGT TCC AAA GGC TA-3′; and 3) *Mertk^–/–^* primer: 5′-ATC AGC AGA CTC TGT TCC AC-3′. PCR products were resolved on 2% agarose gels prepared in Tris-borate–EDTA (TBE) buffer and visualized by ethidium bromide staining.

### 2.5 Method details

#### 2.5.1 Treatment with dexamethasone (PZN-08704344)

Dexamethasone was purchased from JenaPharm with stock concentration of 4mg ml ^-1^, (Catalog No.PZN-08704344; mibe GmbH, Brehna, Germany). The compound was diluted in phosphate-buffered saline (PBS) and administered intraperitoneally at a dose of 2.5mg/kg, with the dose adjusted according to body weight, every 48 hours (h) following infection of mice with 2 × 10^4^ PFU of LCMV-Docile.

#### 2.5.2 Flow cytometry and intracellular staining

Spleen and liver tissues were mechanically dissociated in FACS buffer to generate single-cell suspensions. Cells were stained for 30 min at 4 °C with the following monoclonal Abs: mouse anti-CD11b (clone 53–6.7, eBioscience), anti-CD11c (clone 53–6.7, eBioscience), anti-CD8 (clone 53–6.7, eBioscience), anti-KLRG1 (clone 1D4B), anti-CD44 (clone 1B11, BD Biosciences), anti-CD62L (BD Biosciences), anti-CD127 (BD Biosciences), and anti–PD-1(BD Biosciences). For blood samples, erythrocytes were lysed using 1 ml BD FACS Lysing Solution (BD Biosciences), followed by one wash with FACS buffer. Cells were then stained and analyzed by flow cytometry as described for spleen and liver samples. For intracellular cytokine analysis, splenocytes were fixed in 2% formaldehyde (in PBS) for 10 min at room temperature, permeabilized with 1% saponin, and stained with anti–IL-10(eBioscience, Waltham, MA, USA). After staining, cells were washed with saponin-containing buffer and analyzed by flow cytometry. Data were processed using FlowJo v10.7.2, and mean fluorescence intensity (MFI) values were quantified.

#### 2.5.3 Tetramers

GP33-specific CD8^+^ T cells were identified using allophycocyanin-labelled MHC class I tetramers (GP33/H-2D^b^) obtained from the NIH Tetramer Facility (Bethesda, MD, USA). Single-cell suspensions were incubated with tetramers for 15 min at 37 °C, followed by surface staining with anti-CD8 (clone 53–6.7) for 30 min at 4 °C. For blood samples, erythrocytes were lysed using 1 ml BD FACS Lysing Solution (BD Biosciences), washed once with FACS buffer, and processed for tetramer and CD8 staining as described above. Absolute numbers of GP33-specific CD8^+^ T cells were quantified by flow cytometry using fluorescent counting beads (BD Biosciences). Data were processed using FlowJo v10.7.2, and mean fluorescence intensity (MFI) values were quantified.

#### 2.5.4 Statistical analysis

All statistical analyses were performed using GraphPad Prism (version 10.6.1). Data are presented as mean ± standard error of the mean (SEM). Where indicated, each symbol represents an individual mouse and bars denote mean ± SEM. Differences among multiple groups were assessed using ordinary two-way ANOVA with genotype and treatment as factors, followed by Sidak’s multiple-comparison test. Comparisons between two groups were performed using unpaired two-tailed Student’s *t*-tests. To complement the formal statistical analyses, estimation plots were generated for the dexamethasone experiments. These visualizations display the raw data distribution, effect sizes, and corresponding 95% confidence intervals, thereby improving transparency and aiding data interpretation. Specifically, the estimation plots show the mean difference between dexamethasone treatment groups and are presented as graphical representations of the data rather than as the sole basis for statistical inference. Prism automatically aligned the zero-reference point and scaled the effect-size axis according to its estimation-plot framework; no manual adjustments were applied. P < 0.05; P < 0.01; *P* < 0.001; P < 0.0001; ns, not significant.

## 3 Results

### 3.1 MerTK dependency underlies dexamethasone-induced impairment of CD8^+^ T cell immunity in chronic viral infection

Using VSV, we previously showed that MerTK controls innate immunity and that dexamethasone promotes MerTK-dependent inhibitory signalling, severely suppressing antiviral responses and promoting widespread viral dissemination. These effects were largely absent in MerTK-deficient mice, highlighting MerTK as a key enforcer of GC-mediated immunosuppression. Likewise, our recent work in chronic LCMV infection shows that MerTK drives suppression of antiviral CD8^+^ T cell immunity and worsens disease, whereas *Mertk^–/–^* mice achieve efficient viral control. Based on these insights, we asked whether the dexamethasone–MerTK axis also shapes antiviral CD8^+^ T cell responses during chronic infection. To test this, we infected wild-type (*Mertk^+/+^*) and MerTK-deficient (*Mertk^–/–^*) mice with 2×10^4^ PFU of LCMV Docile, treated with PBS or dexamethasone on days 2, 4, and 6, and immune responses were evaluated on day 8 (**Figure 1A**). This infection dose reliably establishes chronic infection while permitting evaluation of antiviral immune responses and treatment effects. Compared with PBS treatment, dexamethasone markedly reduced total splenic CD8^+^ T cell counts in *Mertk^+/+^*, an effect largely abolished in *Mertk^–/–^* animals (**Figure 1B**). Likewise, the percentage of CD8^+^ T cells in blood, spleen, and liver were significantly reduced in dexamethasone-treated *Mertk^+/+^* mice (**Figure 1C**). Of note, MerTK deficiency substantially abrogated this dexamethasone-strengthened immune suppression, resulting in consistently higher CD8^+^ T cell levels in *Mertk^–/–^* mice across both intervention arms (**Figures 1B, C**). Estimation plot analysis confirmed minimal overlap in cell distributions between genotypes. Effect size analysis revealed mean differences of −17.30 (blood), −23.78 (spleen), and −17.73 (liver) (*Mertk^–/–^*) minus (*Mertk^+/+^*) (**Figure 1D**; **Supplementary Figure 1**), with narrow 95% confidence intervals entirely below zero. Together, these tissue-wide effects suggest that MerTK is necessary for dexamethasone-driven suppression of CD8^+^ T cell responses.

**Figure 1:**
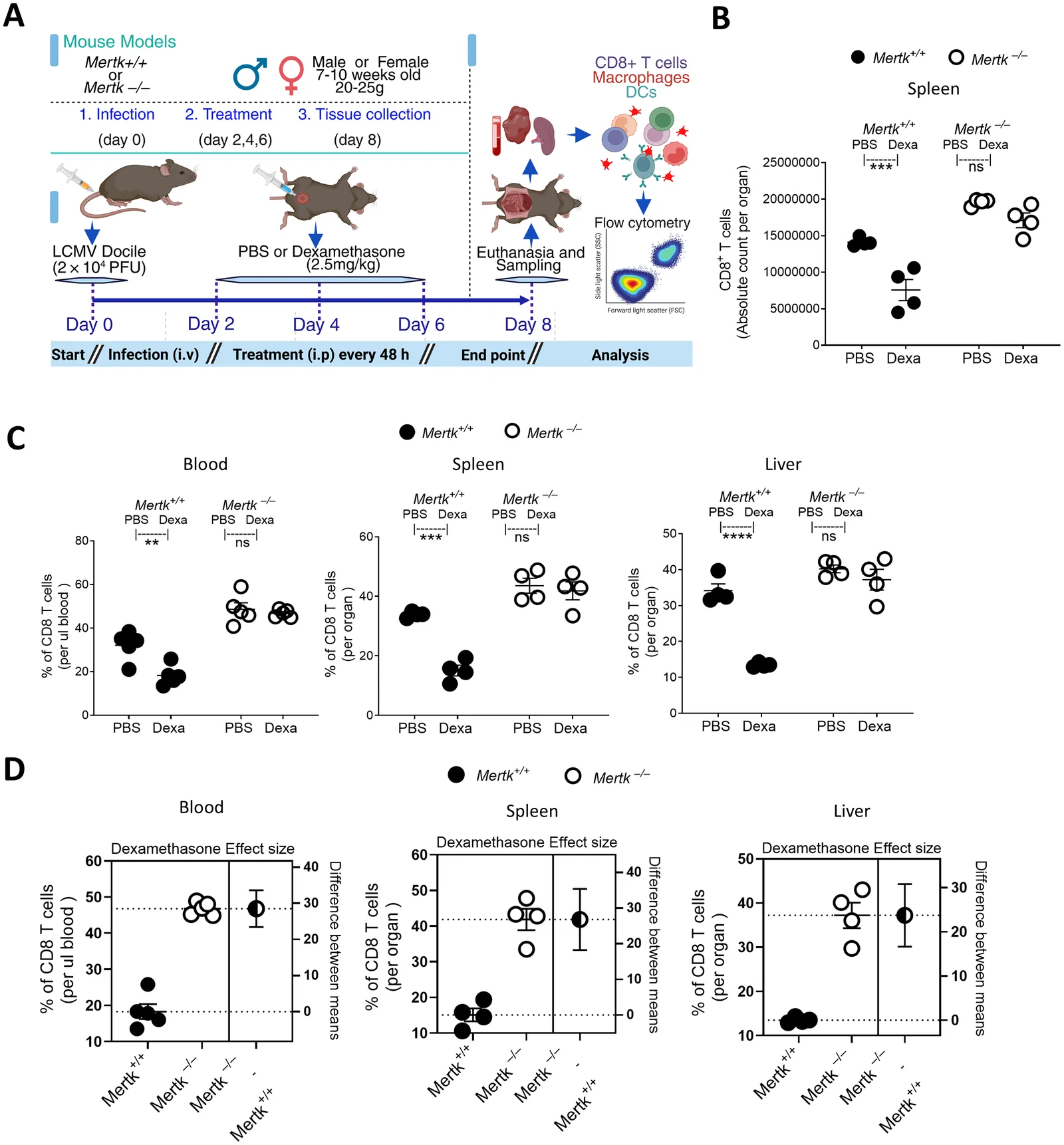
MerTK dependency underlies dexamethasone-induced impairment of CD8^+^ T cell immunity in chronic viral infection. **(A)** Experimental design. Wild-type (*Mertk+/+)* and MerTK-deficient (*Mertk*−/−) mice were infected intravenously (i.v.) with 2 × 10^4^ PFU LCMV Docile (day 0) and subsequently treated with phosphate-buffered saline (PBS; 100 µl/mouse) or dexamethasone (Dexa; 2.5mg/kg) administered intraperitoneally (i.p.) every 48 hours (h) on days 2, 4, 6. Mice were euthanized on day 8 post-infection, the tissues collected and analyzed by flowcytometry or viral titres determined by plague forming assay. Experimental design citation: Created in BioRender. Adomati, T. (2026). Available at: https://BioRender.com/u23oh0k **(B)** Absolute numbers of splenic CD8^+^ T cells in *Mertk*+/+ and *Mertk*−/− mice (n = 4 per group). Each symbol represents an individual mouse; bars indicate mean ± SEM. Statistical significance was assessed using ordinary two-way ANOVA with genotype and treatment as factors, followed by Šídák’s multiple-comparisons test. Genotype effect, F(1, 12) = 69.26, P<0.0001; treatment effect, F(1, 12) = 25.17, P = 0.0003; genotype × treatment interaction, F(1, 12) = 5.142, P = 0.0426. **(C)** Frequencies of CD8^+^ T cells in blood, spleen, and liver of *Mertk*+/+ and *Mertk*−/− mice (n = 5 per group). Each symbol represents one mouse; bars show mean ± SEM. Two-way ANOVA (genotype × treatment) with Šídák’s multiple-comparisons test. Blood: genotype effect, F(1, 16) = 89.92, P < 0.0001; treatment effect, F(1, 16) = 10.88, P = 0.0045; genotype × treatment interaction, F(1, 16) = 6.367, P = 0.0226. Spleen: genotype effect, F(1, 12) = 70.80, P< 0.0001; treatment effect, F(1, 12) = 22.43, P = 0.0005; genotype × treatment interaction, F(1, 12) = 15.52, P = 0.0020. Liver: genotype effect, F(1, 12) = 68.03, P<0.0001; treatment effect, F(1, 12) = 43.19, P< 0.0001; genotype × treatment interaction, F(1, 12) = 23.97, P = 0.0004. **(D)** Frequencies of antigen-specific CD8^+^ T cells in blood, spleen, and liver of *Mertk*+/+ and *Mertk*−/− mice (n = 5 per group). Each symbol represents one mouse; bars show mean ± SEM. Significance was determined by an unpaired two-tailed Student’s t-test. Dexamethasone treatment effect sizes, Blood: P< 0.0001. Spleen: P< 0.0003. Liver: P< 0.0002. ns = not significant. *P< 0.05; **P < 0.01; 757 ***P < 0.001; ****P < 0.0001.

### 3.2 Dexamethasone–MerTK interactions drive profound suppression of LCMV-specific CD8^+^ T cell responses

Chronic viral infections such as HIV, HBV, HCV, and the murine pathogen LCMV are classical settings, in which persistent antigen exposure drives sustained suppression of host immunity (Rehermann and Nascimbeni, 2005; Virgin et al., 2009). Within these environments, antiviral T cell responses are often hampered and fail to control viral replication, allowing long-term viral persistence. We next examined how dexamethasone re-shapes virus-specific CD8^+^ T cell responses, building on our recent insights into MerTK-dependent immunoregulatory circuits operating during chronic LCMV infection. In this setting, PBS-treated *Mertk^–/–^* mice exhibited elevated virus-specific CD8^+^ T cell responses compared to wild type controls (**Figure 2A**; **Supplementary Figure 2**). Dexa treatment caused a pronounced reduction in both the percentage and absolute count of LCMV-specific CD8^+^ T cells in *Mertk^+/+^* mice, compared with PBS-treated controls (**Figure 2A**; **Supplementary Figure 2**). In contrast, *Mertk^–/–^* mice were largely resistant to this dexamethasone-induced CD8^+^ T cell impairment, maintaining robust LCMV-specific responses regardless of treatment condition (**Figure 2A** and **Supplementary Figure 2**). Longitudinal analysis of blood samples further revealed that the percentage of virus-specific CD8^+^ T cells remained significantly diminished at all examined time points in dexamethasone-treated *Mertk^+/+^* mice, whereas *Mertk^–/–^* mice maintained higher LCMV-specific CD8^+^ T cell responses across multiple time points (**Figure 2B**). Moreover, the number of virus specific CD8^+^ T cells (**Figure 2C**), and total CD8^+^ T cells (**Figure 2D**) likewise remained higher in *Mertk^–/–^* mice. Overall, these findings position MerTK as a key element of GC-mediated CD8^+^ T cell suppression with the potential to worsen virus-specific responses in chronic infection.

**Figure 2:**
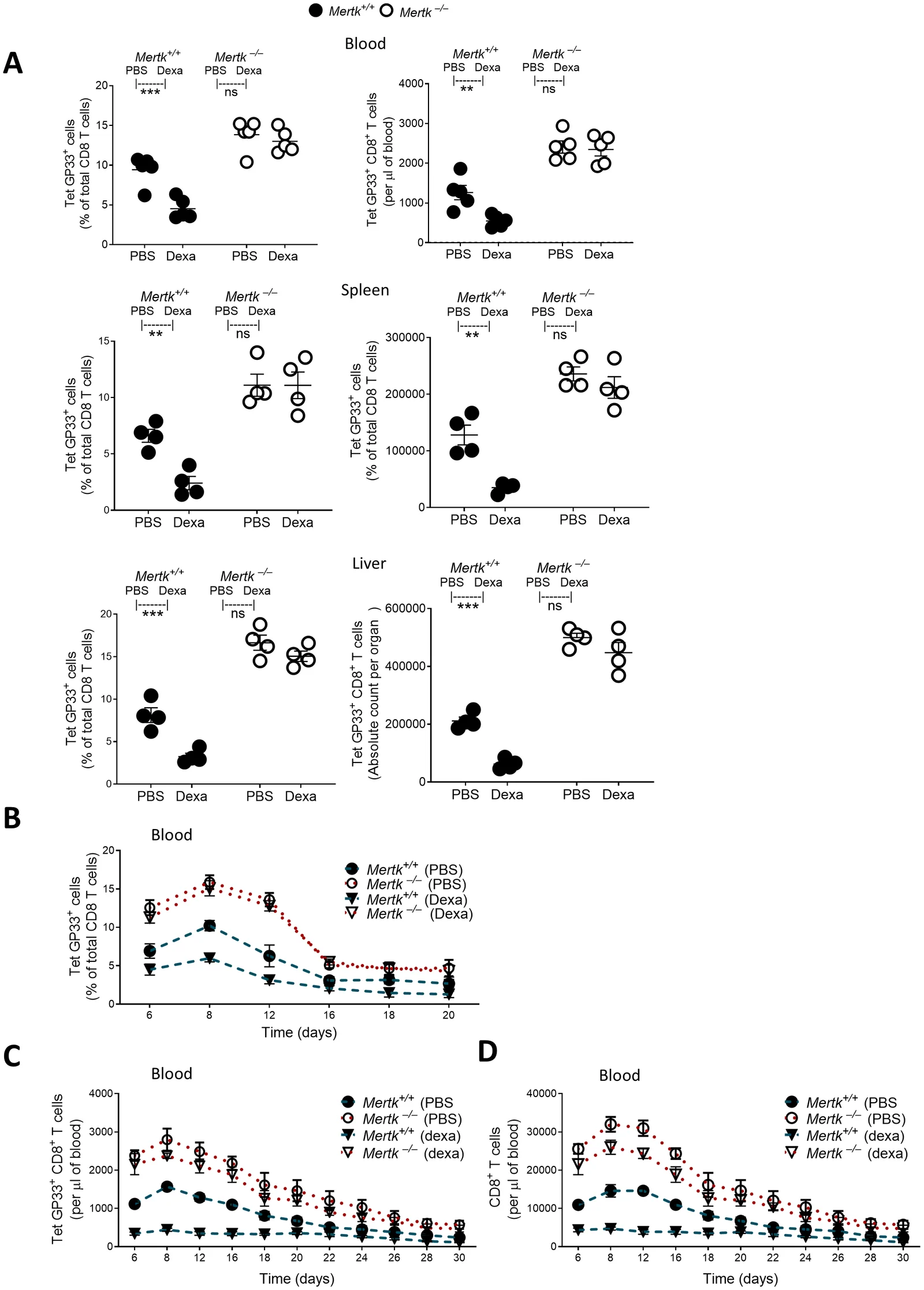
Dexamethasone–MerTK interactions drive profound suppression of LCMV-specific CD8^+^ T cell responses. **(A)**: Percentage and number of Tet-GP33^+^ CD8^+^ T cells in the blood, spleen, and liver of *Mertk^+/+^* or *Mertk^–/–^* mice (n = 5 per group). Each symbol represents one mouse; bars show mean ± SEM. Two-way ANOVA (genotype × treatment) with Šídák’s multiple-comparisons test. Blood (Percentage): genotype effect, F(1, 16) = 74.62, P < 0.0001; treatment effect, F(1, 16) = 14.87, P = 0.0014; genotype × treatment interaction, F(1, 16) = 7.484, P = 0.0147. Blood (Number): genotype effect, F(1, 16) = 100.2, P < 0.0001; treatment effect, F(1, 16) = 6.983, P = 0.0177; genotype × treatment interaction, F(1, 16) = 4.956, P = 0.04407. Spleen (Percentage): genotype effect, F(1, 12) = 56.97, P< 0.0001; treatment effect, F(1, 12) = 5.831, P = 0.0326; genotype × treatment interaction, F(1, 12) = 5.775, P = 0.0333. Spleen (Number): genotype effect, F(1, 12) = 97.80, P< 0.0001; treatment effect, F(1, 12) = 16.33, P = 0.0016; genotype × treatment interaction, F(1, 12) = 5.702, P = 0.0343. Liver (Percentage): genotype effect, F(1, 12) = 198.8, P<0.0001; treatment effect, F(1, 12) = 20.16, P = 0.0007; genotype × treatment interaction, F(1, 12) = 5.197, P = 0.0417. Liver (Number): genotype effect, F(1, 12) = 263.2, P<0.0001; treatment effect, F(1, 12) = 23.47, P = 0.0004; genotype × treatment interaction, F(1, 12) = 5.446, P = 0.0378. **(B)**: Longitudinal analysis of the percentage of Tet-GP33^+^ cells among total CD8^+^ T cells in the blood of *Mertk^+/+^* or *Mertk^–/–^* mice day 6–20 (n = 5 per group). The data are shown as +/- SEM. **(C)**: Longitudinal analysis of the percentage of Tet-GP33^+^ CD8^+^ T cells per µL of blood in *Mertk^+/+^* or *Mertk^–/–^* mice day 6–30 (n = 5 per group). The data are shown as +/- SEM. **(D)**: Longitudinal analysis of Total CD8^+^ T cell count per µL of blood in *Mertk^+/+^* or *Mertk^–/–^* mice day 6–30 (n = 5 per group). The data are shown as +/- SEM. *p < 0.05; **p < 0.01; ***p < 0.001; ****p < 0.0001 (. ns, not significant.

### 3.3 MerTK loss disrupts dexamethasone-induced CD8^+^ T cell functional programs

Next, we assessed how the interplay between dexamethasone and MerTK influences CD8^+^ T cell activation and guides their differentiation fate. In *Mertk^+/+^* mice, dexamethasone markedly reduced the number of KLRG1^+^ CD8^+^ T cells indicating impaired effector-like differentiation (**Figure 3A**), whereas *Mertk^–/–^* mice exhibited increased frequencies of KLRG1^+^ CD8^+^ T cells in a treatment-independent manner indicating that MerTK deficiency promotes the acquisition of an effector-like phenotype and confers resistance to the suppressive effects of dexamethasone on CD8^+^ T cell effector development (**Figure 3A**). CD44^+^ CD8^+^ T cells represent an activated population associated with enhanced effector function and memory potential. In Mertk*^+/+^* mice, dexamethasone treatment markedly reduced the frequency of CD44^+^ CD8^+^ T cells across the analyzed organs, suggesting impaired activation, diminished effector function, and reduced memory potential (**Figure 3B**). In contrast, *Mertk^–/–^* mice sustained markedly higher frequencies of CD44^+^ CD8^+^ T cells under both treatment conditions, demonstrating that MerTK deficiency preserves core CD8^+^ T cell activation and maturation pathways and confers resistance to dexamethasone-mediated inhibition of effector development (**Figure 3B**). CD62L, the lymph node–homing receptor expressed by naïve and central-memory CD8^+^ T cells but downregulated as cells transition into effector states, was markedly elevated by dexamethasone in *Mertk^+/+^* mice, consistent with maintenance of a naïve/central-memory–like phenotype, whereas reduced levels were observed in *Mertk^–/–^* mice, indicative of the acquisition of a more effector-like state (**Figure 3C**). Given the importance of CD127 in T cell survival and lineage commitment, we next assessed its expression. Dexa reduced CD127 expression in *Mertk^+/+^* mice, whereas MerTK-deficient mice displayed a more effector-like profile and were resistant to this effect, as shown by their maintenance of higher CD127 levels (**Figure 3D**). Taken together, these findings reveal that GC-induced restraint of CD8^+^ T cell activation and fate decisions is MerTK-dependent.

**Figure 3:**
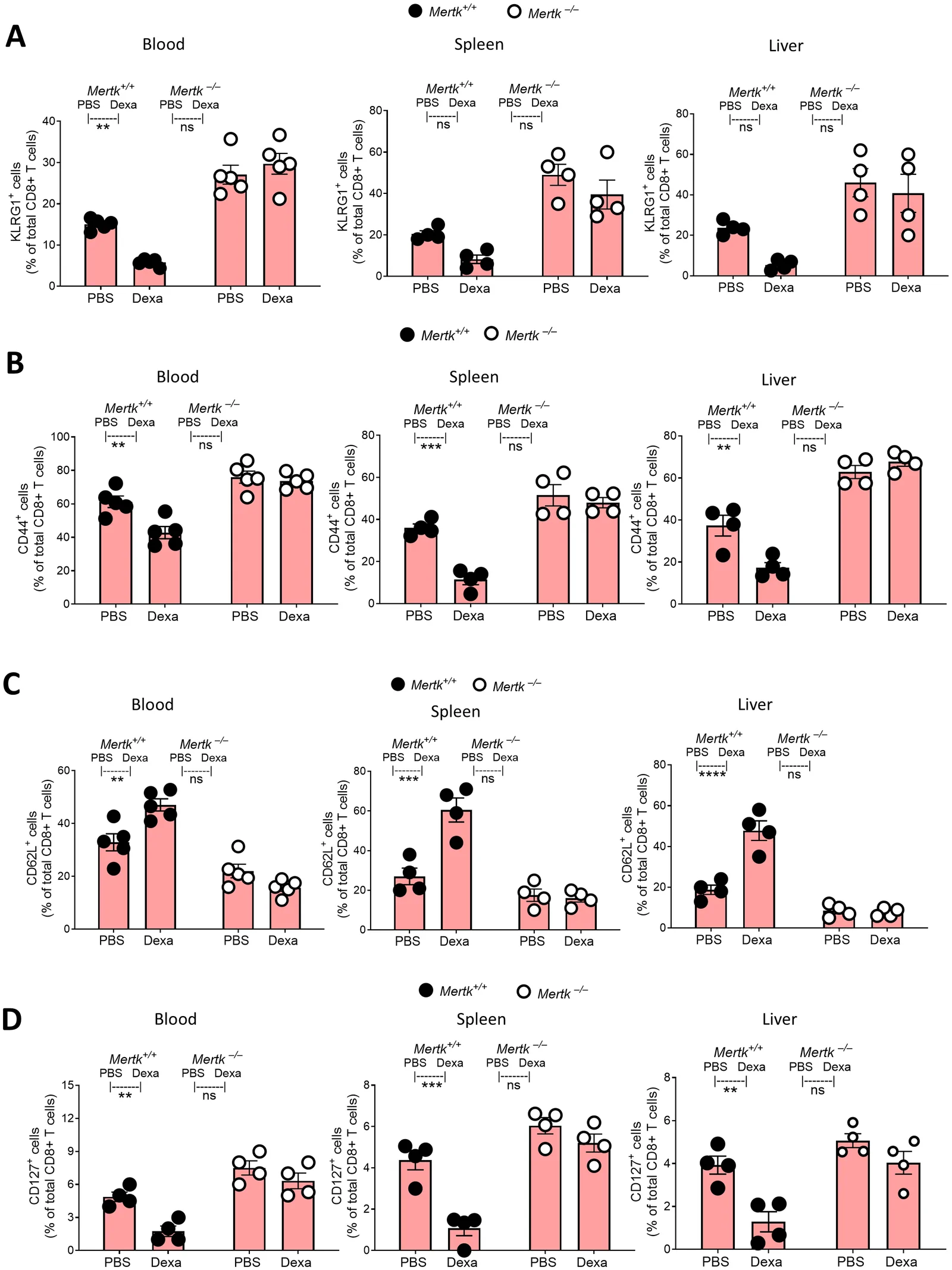
MerTK loss counteracts dexamethasone-induced restraint of the CD8^+^ T cell functional programs. **(A)**: Percentage of KLRG1^+^ CD8^+^ T cells in the blood, spleen, and liver of *Mertk^+/+^* or *Mertk^–/–^* mice (n =4–5 per group). Each symbol represents one mouse; bars show mean ± SEM. Two-way ANOVA (genotype × treatment) with Šídák’s multiple-comparisons test. Blood: genotype effect, F(1, 16) = 106.6, P < 0.0001; treatment effect, F(1, 16) = 3.641, P = 0.0745; genotype × treatment interaction, F(1, 16) = 11.74, P = 0.0035. Spleen: genotype effect, F(1, 12) = 43.95, P< 0.0001; treatment effect, F(1, 12) = 5.824, P = 0.0327; genotype × treatment interaction, F(1, 12) = 0.09310, P = 0.7655. Liver: genotype effect, F(1, 12) = 23.38, P = 0.0004; treatment effect, F(1, 12) = 3.925, P = 0.00709; genotype × treatment interaction, F(1, 12) = 1.210, P = 0.2930. **(B)**: Percentage of CD44^+^ CD8^+^ T cells in the blood, spleen, and liver of *Mertk^+/+^* or *Mertk^–/–^* mice (n =4–5 per group). Blood: genotype effect, F(1, 16) = 48.59, P < 0.0001; treatment effect, F(1, 16) = 10.12, P = 0.0058; genotype × treatment interaction, F(1, 16) = 6.043, P = 0.0257. Spleen: genotype effect, F(1, 12) = 65.90, P< 0.0001; treatment effect, F(1, 12) = 19.18, P = 0.0009; genotype × treatment interaction, F(1, 12) = 10.75, P = 0.0066. Liver: genotype effect, F(1, 12) = 130.2, P<0.0001; treatment effect, F(1, 12) = 5.120, P< 0.0430; genotype × treatment interaction, F(1, 12) = 14.01, P = 0.0028. **(C)**: Percentage of CD62L^+^ CD8^+^ T cells in the blood, spleen, and liver of *Mertk^+/+^* or *Mertk^–/–^* mice (n =4–5 per group). Blood: genotype effect, F(1, 16) = 74.17, P < 0.0001; treatment effect, F(1, 16) = 2.557, P = 0.1294; genotype × treatment interaction, F(1, 16) = 17.48, P = 0.0007. Spleen: genotype effect, F(1, 12) = 42.99, P< 0.0001; treatment effect, F(1, 12) = 15.10, P = 0.0022; genotype × treatment interaction, F(1, 12) = 18.06, P = 0.0011. Liver: genotype effect, F(1, 12) = 79.06, P<0.0001; treatment effect, F(1, 12) = 24.99, P = 0.0003; genotype × treatment interaction, F(1, 12) = 27.71, P = 0.0002. **(D)**: Percentage of CD127^+^ CD8^+^ T cells in the blood, spleen, and liver of *Mertk^+/+^* or *Mertk^–/–^* mice (n = 4–5 per group). Blood: genotype effect, F(1, 12) = 38.87, P < 0.0001; treatment effect, F(1, 12) = 13.86, P = 0.0029; genotype × treatment interaction, F(1, 16) = 2.851, P = 0.1171. Spleen: genotype effect, F(1, 12) = 48.12, P< 0.0001; treatment effect, F(1, 12) = 24.59, P = 0.0003; genotype × treatment interaction, F(1, 12) = 8.702, P = 0.0121. Liver: genotype effect, F(1, 12) = 19.21, P = 0.0009; treatment effect, F(1, 12) = 17.14, P< 0.0014; genotype × treatment interaction, F(1, 12) = 3.307, P = 0.0940.*p < 0.05; **p < 0.01; ***p < 0.001; ****p < 0.0001. ns, not significant.

### 3.4 MerTK and dexamethasone cooperatively enhance IL-10/PD-1 expression and worsen LCMV infection

LCMV’s rapid replication produces a very high early antigen load that initially drives a strong virus-specific CD8^+^ T cell expansion. However, prolonged stimulation ultimately causes T cell exhaustion and loss of function (Moskophidis et al., 1993; Zajac et al., 1998). To prevent excessive immune-mediated damage, the body relies on regulatory mechanisms. IL-10, produced by multiple immune cell types, suppresses T cell activation and effector function, acting as a central brake on inflammation (Kamanaka et al., 2006; Moore et al., 2001). PD-1, expressed on activated T cells, further restricts tissue damage and restrains autoreactive responses by attenuating T-cell receptor signalling and limiting effector activity (Sharpe and Pauken, 2018). In the VSV model, we previously demonstrated that dexamethasone promotes MerTK expression on APCs thereby enhancing MerTK-dependent IL-10 and TGF-β production. This immunoregulatory shift suppresses antiviral cytokine responses and drives increased viral replication in *Mertk^+/+^* mice, a phenotype not observed in *Mertk^–/–^* animals. Similarly, during LCMV infection, in dead cell-dependent manner, MerTK drives the production of IL-10 and TGF-β in APCs and induces PD-1 expression on CD8^+^ T cells, thereby suppressing antiviral defence. Consistent with this mechanism, dexamethasone-treated Mertk*^+/+^* mice showed a sharp increase in APC-derived IL-10 production (**Figure 4A**) and elevated PD-1 expression on both LCMV-specific and total CD8^+^ T cells (**Figure 4B**), whereas both effects were largely reduced in MerTK-deficient mice regardless of therapeutic intervention (**Figures 4A, B**). Given that dexamethasone restrains CD8^+^ T-cell immunity through a MerTK-dependent pathway, we next examined its impact on LCMV replication. Dexamethasone treatment dramatically accelerated viral replication in *Mertk^+/+^* mice, resulting in widespread viral dissemination across all organs examined (**Figure 4C**). As predicted, dexamethasone had a reduced effect in *Mertk^–/–^* mice under identical conditions (**Figure 4C**). These findings mirror our prior results in the VSV model, where dexamethasone and MerTK jointly heightened viral infection, indicating a shared underlying mechanism in which dexamethasone enhances MerTK-mediated suppression of antiviral immunity. Thus, dexamethasone likely exerts its immunosuppressive effects by engaging MerTK-dependent inhibitory pathways, ultimately worsening chronic LCMV infections.

**Figure 4:**
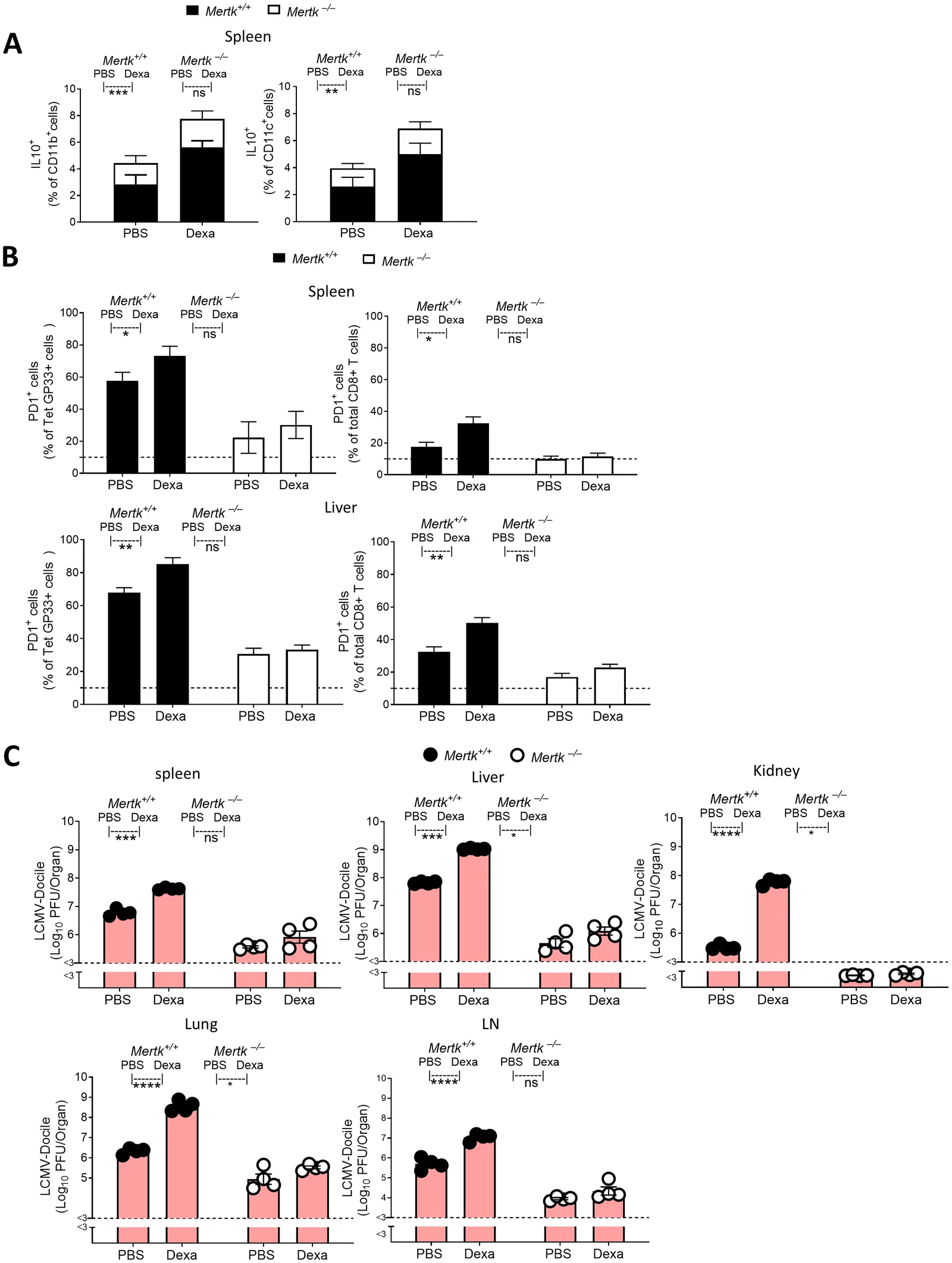
MerTK and dexamethasone cooperatively enhance IL-10/PD-1 expression and worsen LCMV infection. **(A)**: Percentage of IL-10^+^ CD11b^+^ macrophages and IL-10^+^ CD11c^+^ dendritic cells in the spleens of *Mertk^+/+^* or *Mertk^–/–^* mice (n = 3 per group). Each bar show mean ± SEM. Two-way ANOVA (genotype × treatment) with Šídák’s multiple-comparisons test. Spleen (IL-10^+^ CD11b^+^ macrophages): genotype effect, F(1, 8) = 36.98, P = 0.0003; treatment effect, F(1, 8) = 23.35, P = 0.0013; genotype × treatment interaction, F(1, 8) = 10.91, P = 0.0108. Spleen (IL-10^+^ CD11c^+^ dendritic cells): genotype effect, F(1, 8) = 36.98, P = 0.0003; treatment effect, F(1, 8) = 17.06, P = 0.0033; genotype × treatment interaction, F(1, 8) = 6.772, P = 0.0315. **(B)**: Percentage of PD-1^+^ Tet-GP33^+^ CD8^+^ T cells and PD-1^+^ cells among total CD8^+^ T cells in the spleens and livers of *Mertk^+/+^* or *Mertk^–/–^* mice (n = 4 per group). Spleen (PD-1^+^ Tet-GP33^+^ CD8^+^ T cells): genotype effect, F(1, 12) = 105.0, P<0.0001; treatment effect, F(1, 12) = 9.579, P = 0.0093; genotype × treatment interaction, F(1, 12) = 1.055, P = 0.3246. Spleen (PD-1^+^CD8^+^ T cells): genotype effect, F(1, 12) = 24.34, P = 0.0003; treatment effect, F(1, 12) = 8.143, P = 0.0145; genotype × treatment interaction, F(1, 12) = 5.340, P = 0.0394. Liver (PD-1^+^ Tet-GP33^+^ CD8^+^ T cells): genotype effect, F(1, 12) = 177.8, P<0.0001; treatment effect, F(1, 12) = 9.120, P = 0.0107; genotype × treatment interaction, F(1, 12) = 4.830, P = 0.0483. Liver (PD-1^+^CD8^+^ T cells): genotype effect, F(1, 12) = 66.65, P<0.0001; treatment effect, F(1, 12) = 19.67, P = 0.0008; genotype × treatment interaction, F(1, 12) = 5.182, P = 0.0419. **(C)**: Viral titres in organs of *Mertk^+/+^* or *Mertk^–/–^* mice (n = 4 per group). Spleen: genotype effect, F(1, 12) = 165.8, P<0.0001; treatment effect, F(1, 12) = 27.67, P = 0.0002; genotype × treatment interaction, F(1, 12) = 4.833, P = 0.0483. Liver: genotype effect, F(1, 12) = 585.5, P<0.0001; treatment effect, F(1, 12) = 59.95, P < 0.0001; genotype × treatment interaction, F(1, 12) = 13.57, P = 0.0031. Kidney: genotype effect, F(1, 12) = 433.1, P<0.0001; treatment effect, F(1, 12) = 112.5, P < 0.0001; genotype × treatment interaction, F(1, 12) = 48.42, P < 0.0001. Lung: genotype effect, F(1, 12) = 208.9, P<0.0001; treatment effect, F(1, 12) = 84.43, P <0.0001; genotype × treatment interaction, F(1, 12) = 28.75, P = 0.0002. LN: genotype effect, F(1, 12) = 263.0, P<0.0001; treatment effect, F(1, 12) = 39.27, P < 0.0001; genotype × treatment interaction, F(1, 12) = 11.96, P = 0.0047. *p < 0.05; **p < 0.01; ***p < 0.001; ****p < 0.0001. ns, not significant.

## 4 Discussion

Managing aberrant inflammatory conditions in the context of chronic viral infection remains particularly challenging because clinicians must suppress harmful inflammation without compromising antiviral immune responses. The context-dependent actions of GCs underpin both their therapeutic benefits and their diverse adverse effects, many of which remain mechanistically unresolved or limited in their current understanding. Despite decades of clinical use, their impact on antiviral immunity remains unclear and often controversial, and dexamethasone may paradoxically counteract essential antiviral responses in chronic infection. This uncertainty highlights the need for deeper mechanistic insights into how corticosteroids shape immunity in this setting. Effective control of persistent viral replication requires sustained antiviral activity, yet treatments aimed at suppressing pathological inflammation can inadvertently weaken the very responses needed for viral containment. This creates a delicate balance: insufficient immunosuppression risks tissue damage, whereas excessive suppression may trigger viral reactivation and ultimately worsen disease progression. In the context of SARS-CoV-2 and the widespread use of dexamethasone in critically ill patients, its clinical value is undeniable, but its potential adverse effects in individuals living with chronic viral infections deserve careful attention.

We demonstrate that dexamethasone-treated wild type mice undergoing persistent LCMV infection mounted a profoundly suppressive immune response, with major losses in total and virus-specific CD8^+^ T cells and widespread viral replication. This collapse of antiviral immunity reflects the well-known ability of GCs to dampen T cell function and promote tolerogenic myeloid programs. In contrast, MerTK-deficient mice were largely protected from dexamethasone-induced immunosuppressive effects and, despite identical steroid exposure, maintained robust virus-specific CD8^+^ T cell responses and effective viral control. These findings identify MerTK as a critical mediator of GC-driven immune suppression and demonstrate that its absence uncouples steroids from the collapse of antiviral CD8^+^ T cell immunity. Statistical analyses further confirm that MerTK is required for GC-mediated suppression of CD8^+^ T cell activity. In chronic LCMV model, we showed that the peak of CD8^+^ T cell activation coincides with a surge in apoptotic burden and a marked upregulation of MerTK on APCs. This coincides with the induction of anti-inflammatory molecules such as IL-10, TGF-β, and SOCS1/3 in APCs and inhibitory receptor PD-1 on CD8^+^ T cells thereby suppressing antiviral effector responses and ultimately leading to increased viral replication. Notably, these effects were substantially reduced in MerTK-deficient mice, demonstrating that MerTK is a key driver of this immunoregulatory brake during chronic infection. Although not all inhibitory parameters were measured directly apart from IL-10 and PD-1 in the current study, dexamethasone would be expected to further exacerbate these profiles. Consequently, antiviral effectors such as IFN-γ, TNF-α, perforin, and granzyme B are likely suppressed by dexamethasone in wild type but preserved in *Mertk^–/–^* mice. This raises an important clinical concern: widespread dexamethasone use may dampen essential antiviral CD8^+^ T-cell responses during chronic infection by engaging MerTK-dependent inhibitory pathways.

Furthermore, evidence from both animal models and clinical studies reveals mixed and at times conflicting outcomes regarding how corticosteroid treatment influences viral control and disease progression (Fowler et al., 2017; Henneicke et al., 2014). Beyond promoting osteoblast and chondrocyte apoptosis (Chrysis et al., 2005), dexamethasone can induce cell death in diverse cells via mechanisms such as reduced NF-κB activity, IL-6 and CXCL8 production (Price et al., 2015), NF-κB trans-repression (Sharma and Lichtenstein, 2008), reactive oxygen species (ROS) generation autophagy activation and ER stress (Liu et al., 2018). Building on our previous findings, given dexamethasone’s capacity to promote apoptosis, GC treatment likely expands the apoptotic burden thereby enhancing PS availability, in turn facilitating MerTK mediated inhibitory signalling pathways. Collectively, these observations raise the possibility that GC-induced expansion of the apoptotic burden and CTL-mediated cytolysis may augment MerTK-dependent inhibitory signalling, which could contribute to impaired viral control in wild-type animals. An alternative, non-mutually exclusive explanation is that enveloped viruses may engage apoptotic mimicry through PS exposure and subsequent GAS6/PROS1-MerTK signalling (Acciani et al., 2021; Amara and Mercer, 2015; Brindley, 2021; Carnec et al., 2016). Such interactions have been associated with immunoregulatory effects that could contribute to diminished antiviral immunity. Although LCMV lacks PS on its virions, infection generates alternative sources of PS, including apoptotic cells, PS-bearing extracellular vesicles, and PS^+^ dysfunctional CD8^+^ T cells (Medina et al., 2026), which may collectively promote MerTK signalling. Further studies are warranted to define both the mechanisms underlying this differential response and the broader spectrum of dexamethasone-induced immune alterations influenced by MerTK.

At the level of underlying immune mechanisms, chronic viral infections such as LCMV generate diverse memory CD8^+^ T cell populations residing in lymphoid organs, non-lymphoid tissues, and the circulation, whose surface markers reflect their functional states. The expression of KLRG1, CD127, CD62L, and CD44 tightly reflects CD8^+^ T cell functional state, distinguishing memory precursors from short- and long-lived effector-like cells, terminally differentiated populations, and antigen-experienced subsets with varying degrees of exhaustion (Lucas et al., 2024). Consistently elevated expression of KLRG1, CD44, and CD127 in MerTK-deficient mice across treatment conditions underscores MerTK’s role in steering CD8^+^ T cell differentiation towards a defective effector-like phenotype. MerTK loss favours the accumulation of terminally differentiated cytotoxic cells (KLRG1), antigen-experienced effector-memory–like cells (CD44), and long-lived memory-precursor effector cells (CD127). Conversely, higher CD62L expression observed in dexamethasone-treated *Mertk^+/+^* mice suggests that dexamethasone retains a naïve or central-memory–like CD8^+^ T cell phenotype, while limiting progression into fully differentiated effector T cells, consistent with impaired state.

IL-10 is a key regulator of T cell immunity, with many of its effects mediated indirectly through monocyte-derived macrophages that shape downstream lymphocyte responses (Gabryšová et al., 2014; Moore et al., 2001; Wang et al., 2021). Consistent with our previous findings that IL-10 and PD-1 induction during chronic LCMV infection are MerTK-dependent, dexamethasone-treated wild type mice exhibited robust IL-10 production by APCs. Elevated IL-10 likely suppresses antiviral immunity by reducing antigen presentation, limiting co-stimulation, and restraining IL-12 and type I IFN production, thereby impairing CD8^+^ T cell effector differentiation. In parallel, increased PD-1 expression on both virus-specific and total CD8^+^ T cells indicate enhanced T cell dysfunction. Together, these changes were associated with increased viral replication and dissemination, highlighting MerTK as a central mediator of GC-induced immunosuppression during chronic infection. These findings suggest that the detrimental effects of dexamethasone are largely mediated through promotion of MerTK-dependent inhibitory signalling pathways.

Consistent with previous transcriptomic studies, dexamethasone suppresses genes associated with DC maturation and inflammatory signalling while upregulating immunoregulatory factors, including MerTK and IL-10, that promote immune tolerance (García-González et al., 2017). More recent work has shown that dexamethasone induces MerTK expression and phosphorylation in monocyte-derived DCs, promoting a tolerogenic phenotype with impaired maturation, reduced T-cell stimulatory capacity, and enhanced efferocytosis. MerTK inhibition abolishes dexamethasone-induced efferocytosis and reduces Rab17 expression, implicating MerTK in antigen processing and presentation (Li et al., 2023). These findings are consistent with our observations and support a model in which dexamethasone engages MerTK-dependent inhibitory pathways that contribute to the suppression of antiviral immunity. Notably, genetic ablation of MerTK largely attenuated dexamethasone-induced inhibitory programming and was associated with improved viral control. Collectively, these data suggest that MerTK may represent an important mediator through which dexamethasone reinforces tolerogenic signalling and contributes to impaired viral control during chronic infection.

### 4.1 Study limitations

Several limitations of this study should be acknowledged.

1. Although our findings identify MerTK as a critical mediator of dexamethasone-induced immunosuppression during chronic LCMV infection, the downstream mechanisms linking MerTK signalling to elevated IL-10 production, increased PD-1 expression, and CD8^+^ T cell dysfunction were not directly interrogated.
2. While dexamethasone is known to promote apoptosis and could potentially enhance MerTK-dependent signalling through increased apoptotic cell clearance, we did not directly evaluate apoptotic cell burden or efferocytosis in dexamethasone-treated mice. Consequently, this mechanistic link remains hypothetical.
3. Furthermore, we did not perform transcriptomic or proteomic analyses, limiting a comprehensive assessment of the broader inhibitory networks engaged by dexamethasone.
4. While our data support a role for MerTK-dependent tolerogenic programming, processes such as efferocytosis, antigen presentation, GAS6/PROS1 availability, and alternative immunoregulatory pathways including TGF-β, SOCS proteins, regulatory B cells, and Tregs were not directly examined.
5. In addition, our conclusions are primarily based on a constitutive MerTK-deficient model, and developmental compensation cannot be excluded.
6. Finally, because these studies were performed in a single murine model of chronic viral infection, the extent to which MerTK-dependent GC immunosuppression operates across other chronic infections and in humans remains to be determined.
7. Future studies integrating conditional genetic models, pharmacological inhibition, and systems-level profiling will be important to define the full spectrum of mechanisms by which GCs exploit MerTK signalling to suppress antiviral immunity.

## 5 Conclusion

The severe loss of CD8^+^ T cell immunity and worsened disease observed in LCMV-infected, dexamethasone-treated wild type mice, contrasted with the preserved responses in *Mertk^–/–^*animals, demonstrates that the immunosuppressive effects of dexamethasone are largely MerTK-dependent. These findings reinforce the need for caution when using GCs in the context of persistent viral infections. Notably, GCs induce profound CD8^+^ T cell loss, strengthen MerTK-mediated inhibitory pathways, impair antiviral immunity, and skew differentiation toward dysfunctional effector states, ultimately exacerbating chronic viral infection. Together, these results identify MerTK as a potential therapeutic target not only for modulating inflammation and antiviral responses, but also for mitigating steroid-induced suppression of antiviral immunity. They may further inform the clinical monitoring of patients receiving GCs, while acknowledging important species-specific differences between murine and human systems. In addition, our findings underscore the need to define the broader molecular circuitry through which MerTK integrates and amplifies GC-reinforced inhibitory signalling. Although the present study nominates MerTK as a promising therapeutic target, the conclusions are based on genetic loss-of-function approaches, and future studies employing pharmacological inhibition will be required to establish its therapeutic efficacy and translational potential.

## Data Availability

The raw data supporting the conclusions of this article will be made available by the authors, without undue reservation.
